# Concurrent Associations Between Callous-Unemotional Traits, Moral Disengagement, and Bullying Perpetration in Adolescence

**DOI:** 10.1177/08862605241260007

**Published:** 2024-06-19

**Authors:** Robert Thornberg, Linda Wänström, Björn Sjögren, Tiziana Pozzoli, Gianluca Gini

**Affiliations:** 1 Linköping University, Sweden; 2 University of Padova, Italy

**Keywords:** bullying, moral disengagement, callous-unemotional traits, callousness, uncaringness, unemotionality

## Abstract

The aim of the current study was twofold. The first aim was to examine whether callous-unemotional (CU) traits are directly related to moral disengagement and bullying perpetration as well as whether CU traits are indirectly related to bullying perpetration mediated by moral disengagement among adolescents. The second aim was to examine whether the three distinct dimensions of CU—callousness, uncaringness, and unemotionality—are directly related to moral disengagement and bullying perpetration, as well as whether they are indirectly related to bullying perpetration mediated by moral disengagement among adolescents. Self-report survey data from 706 adolescents (*M*_age_ = 14.5) from 20 schools in Sweden were gathered and analyzed using structural equation modeling. The findings suggest that CU traits were positively and directly linked to bullying perpetration, but also indirectly mediated by moral disengagement. Similarly, callousness and uncaringness showed direct and indirect associations with bullying perpetration, whereas unemotionality was found to be only indirectly associated with bullying perpetration. Unemotionality had the weakest connection to moral disengagement and was not directly related to bullying perpetration, whereas callousness, in particular, but also uncaringness, had stronger connections to moral disengagement and bullying perpetration. In sum, the findings underscore the importance of explicitly integrating moral considerations into endeavors aimed at preventing school bullying among adolescents.

The widespread occurrence of school bullying in Sweden (Bjereld et al., 2020; Friends, 2023), where this study was conducted, and around the world (Cosma et al., 2020) produces psychological harm. Being bullied in school is indeed linked to a higher risk of mental health problems (Li et al., 2024; Ye et al., 2023). Within the wide literature on risk factors for bullying, callous-unemotional (CU) traits and moral disengagement are two promising explanatory constructs that can help shed light on how bullying is enabled in schools despite its immoral and harmful nature (Romera et al., 2019), and despite being recognized as a serious moral transgression among students (Thornberg et al., 2017). In this study, we examined whether CU traits were directly related to moral disengagement and bullying perpetration, as well as whether they were indirectly related to bullying perpetration via moral disengagement in a sample of Swedish school students.

## CU Traits

Individual differences in personality traits contribute to explaining why some adolescents are more prone to engaging in bullying perpetration than others. CU traits, in particular, have been noted in the literature as a risk factor for bullying perpetration (Van Geel et al., 2017) and refer to “a specific affective (absence of guilt, constricted display of emotion) and interpersonal (failure to show empathy, callous use of others for one’s own gain) style” (Fanti et al., 2009, p. 285). Adolescents who score high on CU traits display callousness, a lack of empathy, a lack of remorse and guilt, shallow or deficient affect, and a lack of concern about their performance (Hyde & Dotterer, 2022). These personality traits increase adolescents’ propensity to transgress moral and societal norms and violate the dignity and rights of others (Frick et al., 2014). Research has shown that higher levels of CU traits are associated with greater aggression (Gini et al., 2015; Kimonis et al., 2015; Kokkinos et al., 2022; for a meta-analysis, see Ritchie et al., 2022), including bullying perpetration (Catone et al., 2021; Elits & Bäker, 2024; Fanti et al., 2019; Kimonis et al., 2015; Orue & Calvete, 2019; Yang et al., 2021) among children and adolescents. The positive relationship between CU traits and bullying perpetration has been confirmed in two meta-analyses (Van Geel et al., 2017; Zych et al., 2019).

The CU traits construct is characterized by its multidimensionality, encompassing three distinct subconstructs: (a) *callousness*, denoting a deficiency in empathy and a lack of consideration for the well-being, harm, or suffering of others; (b) *uncaringness*, reflecting a lack of concern for societal rules and a disregard for one’s performance in activities deemed socially significant; and (c) *unemotionality*, indicating a tendency to refrain from openly expressing or displaying one’s emotions (Fanti et al. 2009). Despite the multidimensional characteristics, CU traits have, with some exceptions, been studied as a unidimensional construct. In the majority of these studies, in which all three CU traits were included in the same statistical model, callousness and uncaringness were found to be related to aggressive behaviors: general aggression (Goagoses & Schipper, 2021), bullying perpetration (Ciucci et al., 2014; Fanti et al., 2009; Muñoz et al., 2011; Thornberg & Jungert, 2017), and cyberbullying perpetration (Goagoses et al., 2022; Wright et al., 2019).

In a few studies, callousness was the only CU trait that was associated with aggressive behaviors when all three CU traits were considered: general aggression (Ansel et al., 2015), proactive aggression (Fanti et al., 2009), and bullying perpetration (Goagoses et al., 2022; Wang et al., 2019). Further, unemotionality was consistently unrelated to aggressive behaviors when all three CU traits were considered in the analysis (Ansel et al., 2015; Ciucci et al., 2014; Fanti et al., 2009; Goagoses & Schipper, 2021; Goagoses et al., 2022; Muñoz et al., 2011; Thornberg & Jungert, 2017; Wang et al., 2019; Wright et al., 2019). Thus, callousness and uncaringness—but not unemotionality—seem to contribute to aggressive behaviors, including bullying perpetration.

## Moral Disengagement

According to the social-cognitive theory (Bandura 1999, 2016), knowing the differences between what is right and what is wrong is not sufficient to execute moral action and refrain from immoral action. Moral agency also involves motivational and self-regulatory processes, which are needed to translate moral norms into moral behavior. Distortions in these processes produce gaps between moral standards and behavior. Bandura (1999, 2016) proposes *moral disengagement* as a psychological concept that explains why individuals can harm other people and still feel good about themselves. It refers to cognitive distortions that interfere with moral self-monitoring and self-evaluation through justifying, rationalizing, and explaining away immoral and harmful behaviors, which allow individuals to accept and even engage in such behaviors without thinking they are wrong and experiencing feelings of remorse or guilt. Moral disengagement covers eight mechanisms, such as moral justification, euphemistic labeling, diffusion of responsibility, cognitively distorting or ignoring harmful effects, dehumanization, and blaming the victim (Bandura, 1999, 2016).

A large number of studies have found that moral disengagement is positively associated with bullying perpetration (Bjärehed et al., 2021; Elits & Bäker, 2024; Esposito et al., 2022; Orue & Calvete, 2019; Pozzoli et al., 2016; Romera et al., 2021; Teng et al., 2020; Tolmatcheff et al., 2022), including cyberbullying perpetration (Bussey et al., 2015; Fang et al., 2020; Gao et al., 2020; Luo & Bussey, 2022; Orue & Calvete, 2019; Yang et al., 2021). Thus, in accordance with social-cognitive theory (Bandura, 1999, 2016), empirical evidence shows that students who score higher on moral disengagement are more inclined to engage in bullying perpetration. This link has been confirmed in meta-analyses for both school bullying (Gini et al., 2014; Killer et al., 2019; Luo & Bussey, 2023) and cyberbullying (Chen et al., 2017; Killer et al., 2019; Zhao & Yu, 2021).

## Moral Disengagement as a Mediator Between CU Traits and Bullying Perpetration

CU traits contribute to distorting moral reasoning processes (Northam et al., 2022). They are associated with less empathy, guilt, and prosociality (Waller et al., 2019), and can make children and adolescents more inclined to morally disengage from bullying and other aggressive and antisocial behaviors (Paciello et al., 2020). Accordingly, previous research has found a positive relationship between CU traits and moral disengagement (Elits & Bäker, 2024; Fang et al., 2020; Kokkinos et al., 2022, 2016; Orue & Calvete, 2019; Walters, 2018; Yang et al., 2021; Zhao et al., 2023).

In line with social-cognitive theory (Bandura, 1999, 2016) and in reference to the General Aggression Model (GAM; Anderson & Bushman, 2002), Fang et al. (2020) argue that “personal factors interact with situational factors to influence internal states, which affect aggression . . . Specifically, the GAM claims that CU traits may affect an individual’s propensity to aggression and cyberbullying perpetration by distorting social-cognitive processes” (p. 2). However, only a few studies have examined moral disengagement as a mediator between CU traits and various antisocial and aggressive behaviors, such as bullying perpetration. These studies have found that moral disengagement, at least partially, mediates CU traits’ association with bullying perpetration (Elits & Bäker, 2024), cyberbullying perpetration (Fang et al., 2020), relational aggression (Kokkinos et al., 2016), and externalizing problem behaviors (van Leeuwen et al., 2014; Walters, 2018; Zhao et al., 2023).

Specifically, as previously mentioned, callousness and uncaringness, but not unemotionality, tend to be associated with aggressive behaviors, such as bullying perpetration, when included in the same statistical model (Ciucci et al., 2014; Fanti et al., 2009; Goagoses & Schipper, 2021; Goagoses et al., 2022; Muñoz et al., 2011; Thornberg & Jungert, 2017; Wright et al., 2019). It is possible that higher levels of these two CU traits make adolescents more prone to activate moral disengagement when interpreting and handling various social situations, which, in turn, increases the risk of bullying perpetration and other aggressive behaviors. According to social domain theory, morality refers to conceptions of human welfare, justice and rights, and regulations of actions that harm others (Nucci, 2001). Social-cognitive theory states that moral agency is “the ability to refrain from behaving inhumanely” and to manifest “compassion for the plight of others and efforts to further their well-being, often at personal costs” (Bandura, 2016, pp. 1–2).

With reference to how callousness is conceptualized and how it contradicts the moral conceptions above, moral disengagement is probably a “natural” social-cognitive response to this immoral trait. For example, if an adolescent lacks empathy and consideration for others’ well-being, suffering, and harm, it would be easier and more natural for them to justify their bullying behavior, distort its harmful consequences, and dehumanize and blame the victim. This, in turn, should make adolescents high in callousness more likely to engage in behaviors that harm others, demonstrating a lack of consideration for others’ well-being and suffering. Adopting Arsenio and Lemerise’s (2004) aggression and moral development model, which integrates social information processing (SIP) model and social domain theory, adolescents who score low in empathy and care less about others’ well-being and suffering are probably more inclined to activate and use moral disengagement mechanisms when processing social information in social situations. Callousness challenges morality, and moral disengagement serves callousness to override it. It is, therefore, plausible to hypothesize that moral disengagement, at least partially, mediates the association between callousness and bullying perpetration. In support of the hypothetical link between this dimension of CU traits and moral disengagement, previous studies have found that adolescents with low affective empathy are more inclined to show higher moral disengagement (Haddock & Jimerson, 2017; Kokkinos & Kipritsi, 2018; Zych & Llorent, 2019).

On the other hand, uncaringness reflects a lack of concern for societal rules and a lack of care about how well one performs and what others think about one’s performance in activities deemed socially significant. Therefore, uncaringness could also be understood as a trait that should make individuals more susceptible to obscure morality and significant social conventions, as described by social domain theory (Nucci, 2001), by being more inclined to activate and use moral disengagement in their processing of social information, decision-making, and behavior in social situations (cf., Arsenio & Lemerise, 2004). Similar to callousness, uncaringness also challenges societal rules (including morality) and moral disengagement serves uncaringness to override them. Adolescents high in uncaringness are more likely to use moral disengagement mechanisms to engage in, justify, and explain away their misbehaviors, including bullying perpetration. It is, therefore, plausible to hypothesize that moral disengagement, at least partially, mediates the association between this dimension of CU traits and bullying perpetration.

While greater callousness and uncaringness make individuals less concerned about whether their behaviors harm others and transgress societal norms, unemotionality refers to a deficiency in emotional expression. Unemotionality does not challenge societal rules in general or morality in particular; rather, it is more about not openly expressing or displaying one’s emotions. It is, therefore, and in contrast to callousness and uncaringness, reasonable to assume that this CU trait does not increase the risk of aggressive SIP by activating moral disengagement or increasing the likelihood of bullying others. Accordingly, in addition to our hypothesis that unemotionality is not directly linked to bullying perpetration, we hypothesized that unemotionality would not be indirectly associated with bullying perpetration via moral disengagement as a mediator either.

## The Present Study

The present study was the first to examine, in a single model, the associations between the three CU traits, moral disengagement, and bullying perpetration. The aim was twofold. The first aim was to examine whether CU traits are directly related to moral disengagement and bullying perpetration as well as whether CU traits are indirectly related to bullying perpetration mediated by moral disengagement among adolescents. The second aim was to examine whether callousness, uncaringness, and unemotionality are directly related to moral disengagement and bullying perpetration, as well as whether they are indirectly related to bullying perpetration mediated by moral disengagement among adolescents.

First, we hypothesized that CU traits are directly associated with bullying perpetration, as well as indirectly associated with bullying perpetration mediated by moral disengagement. Second, we hypothesized that callousness and uncaringness are directly associated with bullying perpetration, while unemotionality is unrelated to bullying perpetration. Third, we hypothesized that moral disengagement is directly associated with bullying perpetration. Fourth, we hypothesized that callousness and uncaringness are not only directly linked to bullying perpetration but are also indirectly linked to bullying perpetration mediated by moral disengagement, while unemotionality is expected to be neither directly nor indirectly related to bullying perpetration.

## Method

### Participants and Procedure

A non-probability two-step sampling strategy was carried out. In the first step, we used a purposeful sampling of Swedish schools to recruit schools representing different sociogeographic and socioeconomic positions, which resulted in the inclusion of 20 schools. In the second step, a convenience sampling of students in these schools was conducted. An inclusion criterium was that students were either attending upper elementary school (which usually covers ages 10–14) or secondary school (which usually covers ages 13–19).

The initial sample comprised 1,695 students from the selected schools. Consent letters for parental approval were disseminated to all families, with a parental consent rate of 43%. Students were individually sought for their consent, alongside parental approval. Six adolescents opted not to participate, while nine were excluded from the study due to incomplete questionnaire responses. Thus, the final sample consisted of 706 adolescents (44% reported male gender, 55% reported female gender, and 1% reported “other” gender), whose ages ranged from 10 to 20 (*M* = 14.5, *SD* = 2.85), from various socioeconomic (ranging from lower to upper-middle class) and sociogeographic backgrounds. The majority of participants belonged to the Swedish background, whereas a minority (6%) had a non-Swedish background, meaning they were either born in another country or both of their parents had been born in another country. The participants completed a web-based, anonymous, self-report questionnaire on tablets, computers, or cellphones in their ordinary classroom settings. A trained graduate student in psychology was present in the classrooms to explain the study procedure and to assure participant anonymity by instructing participants to create physical distance by moving away from each other and separating their desks. The procedure lasted approximately about 30 to 40 min in each classroom. Ethical approval for the study was obtained from the Regional Ethical Review Board in Linköping.

### Measures

#### CU Traits

We used a short Swedish version (Thornberg & Jungert, 2017) of the Inventory of Callous-Unemotional Traits (ICU; Frick 2004). The short-ICU includes 12 of the original 24 items to assess CU traits in youth, and it is rated on a four-point scale (0 = “Not at all true,” 1 = “Somewhat true,” 2 = “Very true,” and 3 = “Definitely true”). The items cover three subscales: *callousness* (four items, e.g., “I do not care who I hurt to get what I want,” α = .80); *uncaringness* (three items, e.g., “I care about how well I do at school or work” [reversed], α = .75); and *unemotionality* (five items, e.g., “I do not show my emotions to others,” α = 0.73). Cronbach’s α for the whole scale was .76. A hierarchical CFA resulted in good fit (χ_DWLS_^2^(41) = 78.713, *p* < .001 CFI = .976, RMSEA = .036, SRMR = .048), which indicates that the scale can be used to measure CU traits as either a global construct or by its three dimensions; callousness, uncaringness, and unemotionality.

#### Moral Disengagement in Bullying

To assess participants’ tendencies to morally disengage from bullying others, they were asked to respond to four bullying vignettes (hypothetical scenarios) that represented verbal bullying, because this has been found to be the most common form of school bullying (Craig et al., 2009; Man et al., 2022), including in Sweden (Friends, 2023): “Pretend that you are a person who is teasing others. Here are four short stories that we want you to identify yourself with. We want you to pretend to be a person who teases others and who is more popular, powerful, and stronger than the person you are teasing. Don’t worry about whether you have ever done any of these things or not. Just imagine in each story that you are the one who is doing it.” The vignettes represented four different situational characteristics of bullying. Participants were asked to imagine that: (a) their friends were bullying the victim (peer pressure/conformity); (b) peers were watching and laughing because they thought it was fun (reinforcing bystanders); (c) the victim talked badly about their friends (mean victim); and (d) the victim was nice and kind to all their classmates and they liked the victim (likable victim). Repetition of teasing toward the person was built into each vignette (i.e., “a couple of times a week”).

The participants were asked to fill out a 16-item moral disengagement scale, which represented all eight moral disengagement mechanisms, after each vignette (e.g., “Um, I was just kidding with him/her,” “Well, it’s not my fault because a lot of others are doing it to him/her too,” “Well, the kid has him-/herself to blame,” “It’s no big deal. Nobody gets hurt,” “I’m doing a good thing because I do it for a good reason”). A CFA indicated good fit for a one-factor model, with the exception of the SRMR index (χ_DWLS_^2^[1952] = 4759.686, *p* < .001, CFI = .976, RMSEA = .045, SRMR = .092). A hierarchical CFA, however, indicated good fit (χ_DWLS_^2^ [1944] = 2931,047, *p* < .001, CFI = .992, RMSEA = .027, SRMR = .072), indicating that the scale can be used to measure a global construct. Cronbach’s α reliabilities for the moral disengagement scale in each vignette were between 0.94 and 0.95. Cronbach’s α for all 64 items was 0.98 (for a more detailed description of the vignettes and the follow-up moral disengagement scale, see Thornberg et al., 2020).

#### School Bullying Perpetration

The 11-item self-report School Bullying Perpetration Scale (Bjärehed et al., 2021) was used to assess participants’ engagement in school bullying behaviors. They were asked, “Think about the past three months. How often have one or more students who are stronger, more popular, or more powerful than you are done the following things to you at school?” The items covered physical bullying (e.g., “Pushed the student so that it hurt, or so that he/she fell down”), verbal bullying (e.g., “Teased the student and called him/her mean names”), and relational bullying (e.g., “Spread mean rumors or lies about him/her”). Participants rated how frequently they had done each behavior along a 5-point Likert-type scale with response options 1 = “I have never done it,” 2 = “Only a few times,” 3 = “2 or 3 times a month, 4 = “About once a week,” 5 = “Several times a week.” Cronbach’s α was .88 A one-factor CFA indicated good fit (χ_DWLS_^2^[44] = 28.495, *p* = .000, CFI = 1.000, RMSEA = .000, SRMR = .066).

### Data Analysis Procedure

We used Structural Equation Model (SEM) analyses to analyze direct and indirect associations between CU traits and school bullying, in which indirect associations were calculated as the product of two paths. The DWLS estimator was used, and analyses were conducted in R, version 4.2.3, using the Lavaan package. A CFI > .90, an RMSEA < .08, and an SRMR < .08 indicated adequate model fit, and a CFI > .95 and RMSEA < .05 indicated good model fit (e.g., Browne & Cudeck, 1992; Hu & Bentler, 1999; Van de Schoot et al., 2012). α > .85 indicated strong levels, α > .70 indicated acceptable levels of internal consistencies (Kalkbrenner, 2023).

## Results

### Descriptive Statistics and Intercorrelations

In order to present descriptive statistics for the variables CU traits, callousness, uncaringness, unemotionality, moral disengagement, and school bullying perpetration, we calculated the average scores across all items for all participants and all variables. Descriptive statistics are shown in Table 1 and bivariate correlations are shown in Table 2. As shown, boys scored significantly higher on all study variables. CU traits correlated positively with both moral disengagement and school bullying perpetration. When looked at separately, correlations were higher for callousness and uncaringness. In addition, moral disengagement correlated positively with school bullying perpetration.

**Table 1.** Means and Standard Deviations (*SD*) for all Participants and for Girls and Boys Separately, *p*-values, and Cohen’s *d* from *t*-tests for Girls and Boys for the Study Variables.

Variables	Mean (*SD*)	Mean (*SD*)_boys_	Mean(*SD*)_girls_	*p*-Value	Cohen’s *d*
CU	1.98 (0.42)	2.09 (0.42)	1.88 (0.39)	.000	0.52
Callousness	1.42 (0.59)	1.55 (0.66)	1.31 (0.49)	.000	0.41
Uncaringness	1.83 (0.67)	1.97 (0.81)	1.70 (0.60)	.000	0.40
Unemotionality	2.50 (0.60)	2.58 (0.57)	2.43 (0.62)	.001	0.26
MD	1.88 (0.90)	2.07 (0.95)	1.71 (0.81)	.000	0.41
Bullying	1.30 (0.45)	1.42 (0.55)	1.18 (27)	.000	0.57

*Note.* CU = callous-unemotional traits, MD = moral disengagement.

**Table 2.** Bivariate Correlations for the Study Variables.

Variables	1	2	3	4	5	6
1. CU	—	.61***	.62***	.76***	.29***	.28***
2. Callousness		—	.19***	.15***	.31***	.36***
3. Uncaringness			—	.20***	.21***	.23***
4. Unemotionality				—	.12***	.04*
5. MD					—	.41***
6. Bullying						-

*Note.* CU = callous-unemotional traits, MD = moral disengagement, *p < .05, ***p < .001.

### Structural Equation Models

**Figure 1.**
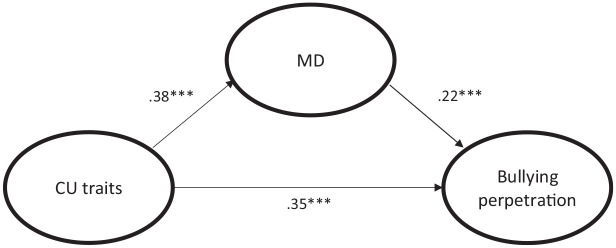
Structural equation model representing the associations between the factors CU traits, MD, and bullying perpetration controlling for gender and age. *Note.* All standardized path coefficients marked with an *** are significant at *p* < .001. Indicators for the three factors, as well as the gender and age variables, are omitted in the graph for simplicity.

Standardized regression path coefficients from a first SEM analysis are shown in Figure 1. We analyzed the direct and indirect associations between CU traits (as a global factor) and school bullying perpetration. Gender and age were used as control variables (not shown in the figure). The model fit statistics indicated good fit (χ_DWLS_^2^[3,732] = 7341.50, *p* < .001, CFI = .973, RMSEA = .037, SRMR = .077). As shown in Figure 1, CU traits were positively and directly associated with moral disengagement and bullying perpetration, and moral disengagement was positively and directly associated with bullying perpetration. In other words, in addition to its direct and positive link with bullying perpetration, CU traits were both directly linked to bullying perpetration and indirectly linked to bullying perpetration mediated by moral disengagement (the indirect path: β = 08, *p* < .001).

Being a boy was positively associated with CU traits (β = .34, *p* < .001), moral disengagement (β = .09, *p* < .001), and bullying perpetration (β = .17, *p* < .001), and age was positively associated with CU traits (β = .11, *p* < .001), moral disengagement (β = .09, *p* < .001), and negatively associated with bullying perpetration (β = −0.19, *p* < .001).

**Figure 2.**
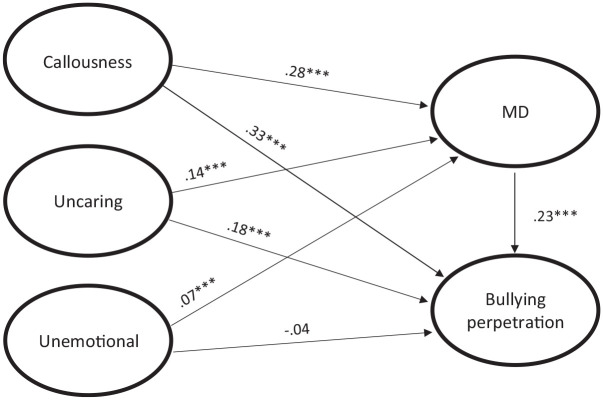
Structural equation model representing the associations between the tree subcategories of CU traits and MD, and the associations between these four variables and bullying perpetration, controlling for gender and age. *Note.* All standardized path coefficients marked with an *** are significant at *p* < .001. Indicators for the five factors, as well as the gender and age variables, are omitted in the graph for simplicity.

The results from a second SEM, in which CU traits were separated into the three factors of callousness, uncaringness, and unemotionality, are shown in Figure 2. The model had good fit (χ_DWLS_^2^ (3724) = 6680.98, *p* < .001, CFI = .978, RMSEA = .034, SRMR = .075). All three CU traits were significantly and positively associated with moral disengagement. The link was strongest between callousness and moral disengagement, and weakest between unemotionality and moral disengagement. Moral disengagement was, in turn, positively associated with bullying. Among the direct paths between CU traits and bullying perpetration, callousness and uncaringness had significant and positive associations. Once again, callousness had the strongest connection. All in all, the model indicated that: (a) callousness, uncaringness, and unemotionality were directly linked to moral disengagement; (b) callousness and uncaringness were directly linked to bullying perpetration; (c) unemotionality was only indirectly linked to bullying perpetration, mediated by moral disengagement (β = .02, *p* < .001). In contrast, callousness and uncaringness were both directly linked to bullying perpetration and indirectly linked to bullying perpetration mediated by moral disengagement (callousness: β = .07, *p* < .001; uncaringness: β = .03, *p* < .001).

We controlled for gender and age in the model. Being a boy was positively associated with callousness (β = .35, *p* < .001), uncaringness (β = .39, *p* < .001), unemotionality (β = .31, *p* < .001), moral disengagement (β = .07, *p* < .01), and bullying perpetration (β = .11, *p* < .001). Age, in turn, was positively associated with callousness (β = .12, *p* < .001), uncaringness (β = .24, *p* < .001), unemotionality (β = .12, *p* < .001), and moral disengagement (β = .08, *p* < .001), and negatively associated with bullying perpetration (β = −.20, *p* < .001). Thus, older adolescents were more inclined to score higher in all three dimensions of CU traits and in moral disengagement, but were less prone to engaging in bullying behaviors.

## Discussion

To our knowledge, the present study was the first to examine whether the CU traits of callousness, uncaringness, and unemotionality were directly associated with bullying perpetration and moral disengagement as well as whether these three dimensions of the CU traits were indirectly associated with bullying perpetration mediated by moral disengagement among adolescents within a single statistical model. The first step was to test a model in which CU traits were included as a global construct. In accordance with our hypothesis, CU traits were positively related to bullying perpetration both directly, and indirectly; mediated by moral disengagement. Our findings support previous research showing that CU traits are positively associated with moral disengagement (Elits & Bäker, 2024; Kokkinos et al., 2016, 2022; Orue & Calvete, 2019; Yang et al., 2021; Zhao et al., 2023) and bullying perpetration (Van Geel et al., 2017; Zych et al., 2019), as well as the positive relationship between moral disengagement and bullying perpetration (Gini et al., 2014; Killer et al., 2019; Luo & Bussey, 2023). Our results also confirm the indirect association between CU traits and bullying perpetration via moral disengagement found in Eilts and Bäker’s (2023) study.

The final step was then to test a model where the global construct of the CU traits was replaced with its three dimensions. In accordance with our hypotheses, the present findings showed that callousness and uncaringness were both directly associated with bullying perpetration and indirectly associated with bullying perpetration mediated by moral disengagement, while unemotionality was not directly associated with bullying perpetration. However, in contrast to our expectations, unemotionality was indirectly associated with bullying perpetration mediated by moral disengagement, but its direct link to moral disengagement was the weakest significant link in the model.

Our findings regarding the direct paths between CU traits and bullying perpetration were expected and in line with other studies showing that callousness and uncaringness—but not unemotionality—are related to general aggression (Goagoses & Schipper, 2021), bullying perpetration (Ciucci et al., 2014; Fanti et al., 2009; Muñoz et al., 2011; Thornberg & Jungert, 2017), and cyberbullying perpetration (Goagoses et al., 2022; Wright et al., 2019). We found that callousness had the strongest direct link to bullying perpetration, which can be compared to a few studies in which callousness was the only CU trait that was found to be significantly related to general aggression (Ansel et al., 2015), proactive aggression (Fanti et al., 2009), and bullying perpetration (Goagoses et al., 2022; Wang et al., 2019).

Callousness and uncaringness were also directly related to moral disengagement, which was expected because callousness refers to a lack of concern about others’ welfare, harm, and suffering, and uncaringness refers to a lack of concern about one’s performance in activities considered socially important. It is reasonable to assume that adolescents who score high on these traits are more inclined to activate moral disengagement mechanisms and engage in antisocial and immoral conduct such as bullying perpetration (see also, Romera et al., 2019).

A possible conceptual explanation for why these two CU traits play a significant role in moral disengagement and bullying perpetration might be that higher callousness (unconcerned about others’ well-being and harm) and uncaringness (unconcerned about societal rules and how others think about one’s performance in socially significant situations) make it easier and more natural to activate and use moral disengagement mechanisms when processing social information. It is, for example, easier for adolescents to justify their bullying, distort its harmful effects, and dehumanize the victim if they do not care about others’ well-being or societal rules and standards. Thus, adolescents high in callousness and uncaringness might have developed a disposition to activate a fast, implicit processing of moral disengagement to serve these CU traits that underpin their aggressive SIP patterns.

From a SIP model perspective (Arsenio & Lemerise, 2004; Crick & Dodge, 1994; Verhoef et al., 2022), their habitual use of moral disengagement can be understood as easily primed hostile schemas that shape their aggressive SIP and actions (Verhoef et al., 2022). Due to its high *accessibility* and *habitual use*, moral disengagement will more likely override these adolescents’ moral structures (learned from and internalized through moral socialization, see Bandura, 2016; Nucci, 2001). Moreover, it will interfere with how these adolescents interpret social cues (e.g., victim blame), formulate social goals (and justify these), raise possible sociomoral concerns (i.e., being less inclined to do that), and evaluate possible actions to take (including how to perceive, justify, and label these actions) and their possible consequences (including being less likely to see how one’s aggressive or inhumane behaviors result in harm and unfairness). Higher levels of callousness and uncaringness and their links to greater moral disengagement would, in turn, make adolescents more likely to select and enact aggressive behaviors such as bullying perpetration (compare with Arsenio & Lemerise, 2004; Crick & Dodge, 1994; Verhoef et al., 2022).

Among the CU traits, callousness had the strongest link to moral disengagement in the present findings, which can be compared with Thornberg and Jungert’s (2017) study, showing that adolescents who scored higher in callousness were less prone to display harm-effect moral reasoning when judging bullying behavior. As Elits and Bäker (2023) put it, “Since people with higher levels of the CU traits do not care about the feelings of others, they are presumably better able to justify harming others or accepting the harm of others” (p. 8). Conceptually, callousness is the CU trait that most clearly and directly challenges and undermines the moral domain in terms of others’ well-being, justice, and rights, and regulations of actions that harm others (see Nucci, 2001), which may explain why this dimension of CU traits had strongest links to both moral disengagement (regarding peer aggression) and bullying perpetration.

The indirect relationship between unemotionality and bullying perpetration via moral disengagement did not correspond to our hypothesis. However, as Thornberg and Jungert (2017) argue, unemotionality refers to a lack of emotional expression, which indicates emotional disengagement, emotional disconnection, and poor emotional awareness of oneself and others. Being unemotional indicates poor emotional intelligence. Thus, a possible explanation for this indirect but weak link in our findings could be that adolescents who are less inclined to “perceive, manage, and reason about emotions within oneself and others” (Kahn et al., 2016, p. 903) might be less capable of recognizing emotions such as distress, sadness, and psychological harm in others, and may therefore be more prone to morally disengage in bullying situations. In this way, unemotionality might contribute to bullying perpetration via moral disengagement. Nevertheless, this CU trait had the weakest connection to moral disengagement and was not directly related to bullying perpetration.

Unemotionality is more about deficits in expressing or displaying one’s emotions in social situations than influencing one’s behaviors toward others and activating moral disengagement to justify one’s aggressive behaviors. In contrast, callousness, in particular, but also uncaringness, had stronger connections to moral disengagement and bullying perpetration, as they collide with morality and societal rules. This indicates their role in undermining adolescents’ moral agency and facilitating aggressive behaviors by making adolescents more prone to activate and use moral disengagement mechanisms, which increases the risk of aggressive SIP and bullying perpetration.

### Limitations

Some limitations of this study should be considered. First, the analyses were based on self-reported data, which are vulnerable to social desirability, perception, and recall biases, but also to the risk of inflated variable associations due to shared method variance. Second, using vignettes and asking students to imagine being someone in a hypothetical scenario who is doing something independently of how they behave in real life in order to measure moral disengagement might be problematized in terms of ecological validity. However, the positive correlations with both CU traits and bullying perpetration in the study indicate good criterion-related validity, and thus external validity, in addition to its high internal consistency. In addition, “consistent with the theoretical basis of moral disengagement, the moral disengagement scale is tailored to the context investigated” (Bussey, 2020, p. 312), which, in the current study, is the context of school bullying.

Third, the cross-sectional design of the current study keeps us from drawing causal conclusions or pinpointing the direction of the relationships between the study variables. For example, it is not clear whether the three CU traits and/or moral disengagement predict bullying perpetration, or whether bullying perpetration predicts the three CU traits and/or moral disengagement. Neither can we determine whether the three CU traits influence moral disengagement, or whether moral disengagement influences the three CU traits. It is also possible that some or all of these associations are reciprocal. Nevertheless, from the theoretical point of view, CU traits are considered to be personality traits (Fanti et al., 2009; Frick et al., 2014; Hyde & Dotterer, 2022), and it would therefore be more likely that they will predict moral disengagement and bullying perpetration rather than the other way around. Most studies that have examined possible links between CU traits (as a global construct), moral disengagement, and various antisocial behaviors have tested models in which moral disengagement mediates the relationship between CU and antisocial behaviors (Paciello et al., 2020). However, alternative models could be examined, including a complex and reciprocal interplay between these constructs (Paciello et al., 2020), which would be in line with the social-cognitive theory of moral disengagement (Bandura, 2016). Therefore, future studies should employ a longitudinal design to examine possible reciprocal associations across the three CU traits, moral disengagement, and bullying perpetration. In addition, multilevel analyses may be conducted in order to separate within-person and between-person variance. Fourth, while efforts were made to include participants from various socioeconomic and sociogeographic backgrounds, the sample may not fully represent the broader adolescent population in Sweden, thereby potentially limiting the generalizability of the findings. Further research involving more diverse and representative samples, both within and beyond Sweden, is necessary to validate and extend the findings of the current study.

### Practical Implications

Despite its limitations, the findings of the current study could have significant practical implications. Specifically, they underscore the importance of explicitly integrating moral considerations into endeavors aimed at preventing bullying. One potential approach to tackle this issue would be to develop strategies that specifically target the reduction of moral disengagement. Educators and school psychologists should engage in open discussions with students regarding moral disengagement, and assist them in recognizing and challenging these harmful mechanisms when they arise in their daily social interactions at school.

Although addressing CU traits in the school environment may not be easy, another approach to preventing and reducing bullying could involve nurturing students’ moral identities (Pozzoli et al., 2016). A strong moral identity not only motivates individuals to act morally but also discourages immoral behavior, as it creates a psychological need to align one’s actions with moral standards (Blasi, 1993). To promote the development of moral identity in schools, Lapsley and Stey (2014) emphasize the importance of fostering a sense of connection and care within the school community. This includes fostering strong attachments between students and teachers, providing opportunities for students to develop self-control and integrity in accordance with moral values, and encouraging engagement in acts of kindness and service.

Additionally, incorporating moral issues into prevention programs can also empower bystanders to actively intervene. One of the primary challenges with “unconcerned bystanders” (Obermann, 2011) is that they often fail to recognize interpersonal harm as a moral problem. Therefore, educational activities should strive to raise awareness about the moral aspects of bullying and emphasize the responsibilities of bystanders. This can be achieved by educating students about the presence of moral disengagement mechanisms and encouraging them to play an active role in addressing bullying situations. Even though boys tended to show higher CU traits, moral disengagement and bullying perpetration than girls, and older students were inclined to score higher in CU traits and moral disengagement but less in bullying perpetration than younger students, the findings underscored that the relationships between the variables were significant when controlling for gender and age. Thus, addressing CU traits (callousness and uncaring in particular) and moral disengagement is important in bullying prevention independently of students’ gender and age.
